# Document-Image Related Visual Sensors and Machine Learning Techniques

**DOI:** 10.3390/s21175849

**Published:** 2021-08-30

**Authors:** Kyandoghere Kyamakya, Ahmad Haj Mosa, Fadi Al Machot, Jean Chamberlain Chedjou

**Affiliations:** 1 Institute for Smart Systems Technologies, Faculty of Technical Sciences Universitaet Klagenfurt, A9020 Klagenfurt, Austria; ahmad.haj.mosa@pwc.com; 2 Research Center Borstel—Leibniz Lung Center, 23845 Borstel, Germany; falmachot@fz-Borstel.de (F.A.M.); Jean.Chedjou@aau.at (J.C.C.)

Document imaging/scanning approaches are essential techniques for digitalizing documents in various real-world contexts, e.g., libraries, office communication, management of workflows, and electronic archiving. Such a digitalization step plays an important role in decreasing costs and increasing the efficiency of document management systems.

Document management systems require document imaging/scanning approaches to convert hard-copy documents/images into digital files. However, document management systems are complex systems consisting of database servers and any document analysis related processes. The term document management refers to the database-supported management of electronic documents. A basic application of document management in the narrower sense is the digital files, in which information from various sources is either extracted or fused and refers to multiple system categories and their interaction in the broader sense.

Furthermore, the added value of such systems arises when documents have to be retrieved and/or analysed after some time due to legal requirements, and such a retrieval/analysis can be avoided or be related to financial penalties that can be significant for the industry. Moreover, costs and efforts can be reduced by retrieving documents. Increasingly, document imaging systems are being used as the base for organizational programs. The completion of tasks, orders, etc., is thus supported in logical and temporal sequences as workflows.

Since the conversion is not merely an image, Optical Character Recognition (OCR) is consecutively involved in recognizing and extracting the information contained in the document images. The documents can then be indexed and the extracted information can be transferred to a document management system for further processing. However, the OCR system does not show promising performance whenever images might be curved, distorted (e.g., by noise, blur, low contrast, and shadow), skewed, or have insufficient resolution, resulting in the loss of valuable image assets for character identification. Particularly hard distortion conditions occur nowadays when document images are acquired by using smart phone cameras. This means that while the image is accessible, the document might, however, not always be clearly readable.

In the state-of-the-art, there are many approaches to overcome the challenges of digital imaging/scanning systems: for example, utilizing self-learning systems with similarity/embedding vectors, neural models, and deep learning. Furthermore, pattern recognition can be used in two ways: (a) to determine the location of a predefined pattern in a larger image area, e.g., in a pick-and-place application where a vision system finds the object or the bar code and transmits the position to a robot; and (b) to focus classification on the nature of the visible object at a given location, e.g., in the case of text recognition where the position of each character is known but where it is necessary to determine which letter or digit is present.

Generally, high quality captured document images are required due to a series of challenges related to the performance of the visual sensors and, for camera-based captures, difficult external environmental conditions encountered during the sensing (image capturing) process. Such document images are mostly hard to read, have low contrast, and are corrupted by various artifacts such as noise, blur, shadows, spot lights, etc., just to name a few. To ensure an acceptable quality of the final document-images that can be perfectly digitalized and involved in various high-level applications based on digital documents, the sensing process must be made much more robust than the raw capture result generated by a purely physical visual sensor. Thus, the physical sensors must be virtually augmented by a series of additional pre-processing and/or post-processing functional blocks, which mostly involve, amongst others, advanced machine learning techniques.

This book emerging from the Special Issue "Document-Image Related Visual Sensors and Machine Learning Techniques” can be viewed as a result of the crucial need for document management systems. Such technologies are being applied in various fields or different domains and parts of the world to address challenges that could not be addressed without the advances made in these technologies. The Special Issue includes nine papers submitted in response to the call for papers. The Special Issue includes impactful papers that present scientific concepts, frameworks, architectures and innovative ideas on sensing technologies and machine-learning techniques to overcome the challenges of document imaging/scanning, test detection, text recognition and documents clustering.

Overall, these papers can be grouped into the following three categories/groups:

- Visual Sensing;
- Document scanning and imaging;
- Document clustering and classification.

## 1. Visual Sensing

In [1], the authors propose a sensing concept for reliably classifying different types of houses. For this challenging endeavour, they propose/introduce a novel convolutional neural network architecture involving multi-channel features extraction. The developed deep-learning model was trained with 600 images, verified with 200 images, and tested with 400 other images. The performance (accuracy, precision, and so on) reached by the proposed CNN model is at least 8% higher than that of the related models from the previous state-of-the-art, which have been involved in the rigorous benchmarking.

The authors of [2] suggest a composite filtering system for using consumer depth cameras at close range. The proposed method comprises three key components which work together to remove various forms of noise. The system is GPU-accelerated and does not use window smoothing. The proposed approach has been tested by using both Kinect v2 and SR300. The results demonstrate promising results and have exceptionally high real-time accuracy, allowing it to be used as a pre-processor for real-time human-computer interaction and real-time 3D reconstruction.

## 2. Document Scanning and Imaging

Given the wide range of image binarization methods available and their various implementations and image types, it is not easy to consider a single standardized threshold approach to be the right option for all images. There is still a lack w.r.t. deciding which binarization methods are prone to increase OCR accuracy. As a result, the concept of using robust combined steps is discussed in the work presented in [3], whereby the benefits of different techniques are integrated/merged though including some recently suggested approaches focusing on entropy filtering and a multi-layered stack of regions. The experimental results obtained for the WEZUT OCR Dataset, a dataset of 176 non-uniformly illuminated text images, clearly confirm both the feasibility and utility of the proposed solution, resulting in substantial improvement in recognition accuracy.

The work in [4] proposes a low-cost scanner for capturing multispectral paper images. Here, the authors modify a sheet-feed scanner by adding an external multispectral light source made up of narrow-band light-emitting diodes to its internal light source (LED). The modification does show promising results, coupled with compactness and low cost. The prototype design can be transformed into a fully functional portable product that can be used for multipurpose document analysis.

## 3. Document Clustering and Classification

In [5], the authors propose a scene text recognition algorithm using a text location correction (TPC) module and an encoder-decoder network (EDN) module. The TPC module converts the slanted text unto a horizontal text, and the EDN module then identifies the content of the flat text. For evaluation, the authors used both the ICDAR2013 and IIIT5K datasets. The experiments and the related evaluation results show promising results, and they additionally show that the proposed approach is capable of recognizing a wide range of odd text. The proposed two network modules improve the precision of abnormal scene text detection according to ablation studies.

The paper [6] introduces a Deep Convolutional Neural Network (DCNN)-based real-time supervised learning strategy for document classification that aims to reduce the influence of negative document image issues such as signatures, labels, logos, and handwritten notices. The authors propose a data augmentation strategy that uses the secondary dataset RVL-CDIP to normalize the imbalanced dataset. DCNN features are extracted using the VGG19 and AlexNet networks that are then fused, optimized, and modified by removing the redundant features using the Pearson correlation coefficient-based technique. The proposed approach is evaluated on the Tobacco dataset, whereby it shows promising classification results using a cubic support vector machine classifier.

In [7], the authors propose a text recognition Convolutional Neural Network (CNN) architecture that is adaptive to text scale to solve this problem. They use multi-stage convolution layers to extract multi-resolution feature maps in order to avoid missing details and to keep the feature size constant. The evaluation of the proposed model is performed using 7152 natural scene images containing texts. The main improvement is to introduce a multiple Region Proposal Network (RPN) to detect texts from different resolution feature maps. The suggested system outperforms the faster R-CNN by more than seven points on the F-score in the conducted experiments. Furthermore, the proposed approach produces findings that are similar to those of other methods. As a result, they have comprehensively tested the efficacy of the proposed approach, especially for text scales.

In [8], the work proposes a clustering approach in Wireless Multimedia Sensor Networks (WMSN). The aim is to overcome the problem of feature extraction from incomplete data. Therefore, the researchers of this work suggest (a) the use of the optimally constructed variational autoencoder networks for feature extraction from incomplete data, (b) improving the clustering output by using the High-Order Fuzzy C-Means algorithm (HOFCM), and (c) recovering the missing data by using low-dimensional latent space of the variational autoencoder. The experiments on different datasets show that the proposed algorithm improves the clustering accuracy for incomplete data and fills in missing features properly.

The research in [9] contributes in detecting and recognizing charts. The proposed system automates the process by using perspective detection and correction. These methods transform a blurred and noisy input into a simple chart that is ready for data extraction. Different models have been tested for classification and detection, e.g., Xception, ResNet152, VGG19, MobileNet, RetinaNet, and Faster Region-Based Convolutional Neural Network (R-CNN). The authors collected 21,099 chart images from Google, Baidu, Yahoo, Bing, AOL, and Sogou for evaluation. The total number of charts’ classes is 13. The obtained results and the evaluation metrics in this work show that chart recognition methods can be applied for real-world applications.
